# More Adverse Events after Osteosyntheses Compared to Arthroplasty in Geriatric Proximal Humeral Fractures Involving Anatomical Neck

**DOI:** 10.3390/jcm10050979

**Published:** 2021-03-02

**Authors:** Felix Porschke, Julia Bockmeyer, Philip-Christian Nolte, Stefan Studier-Fischer, Thorsten Guehring, Marc Schnetzke

**Affiliations:** 1 BG Trauma Center Ludwigshafen at Heidelberg University Hospital, Ludwig-Guttmann-Straße 13, 67071 Ludwigshafen, Germany; j.bockmeyer@gmx.net (J.B.); nolte_philip@yahoo.de (P.-C.N.); stefan.studier-fischer@bgu-ludwigshafen.de (S.S.-F.); marc.schnetzke@atos.de (M.S.); 2 Department of Orthopedic Surgery, Paulinenhilfe, Diakonieklinikum Stuttgart, Rosenbergstraße 38, 70176 Stuttgart, Germany; guehring@uni-heidelberg.de; 3 German Joint Center, Atos Clinic Heidelberg, Bismarckstraße 9-15, 69115 Heidelberg, Germany

**Keywords:** proximal humeral fracture, geriatric, osteosynthesis, ORIF, arthroplasty, complication, revisions, shoulder

## Abstract

The purpose of this study was to compare adverse events and clinical outcomes of geriatric proximal humerus fractures (PHF) involving the anatomical neck (type C according to AO classification) treated with open reduction and internal fixation (ORIF) using locking plate vs. arthroplasty. In this retrospective cohort study, geriatric patients (>64 years) who underwent operative treatment using ORIF or arthroplasty for type C PHFs were included. Complications, revisions and clinical outcomes using Constant Murley Score (CMS) and Disabilities of the Arm, Shoulder and Hand (DASH) Score were assessed and compared between groups. At a mean follow up of 2.7 ± 1.7 years, 59 patients (mean age 75.3 ± 5.5 years) were included. In 31 patients ORIF was performed and 29 patients underwent arthroplasty. Complications and revision surgeries were significantly more frequent after ORIF (32.6% vs. 7.1%, *p* = 0.023 and 29.0% vs. 7.1%, *p* = 0.045). In contrast, clinical outcomes showed no significant differences (DASH 39.9 ± 25.7 vs. 39.25 ± 24.5, *p* = 0.922; CMS 49.7 ± 29.2 vs. 49.4 ± 25.2, *p* = 0.731). ORIF of type C PHFs in geriatric patients results in significantly more complications and revision surgery when compared to arthroplasty. Therefore, osteosynthesis of geriatric intraarticular fractures of the proximal humerus must be critically evaluated.

## 1. Introduction

Proximal humeral fractures (PHF) are common especially in elderly patients [1]. The incidence of PHFs is increasing with age and has risen in recent decades [2]. Finding the right treatment for elderly patients with comorbidities is challenging [3,4]. Particularly in slightly displaced and stable fractures conservative treatment is usually recommended [3,5]. For patients with complex fractures optimal treatment remains controversial. Besides nonoperative treatment, open reduction and internal fixation (ORIF) or arthroplasty are the most popular treatment options [4,6,7,8]. Internal locking-plate fixation has been increasingly used in recent years. Here promising results have been published, but complications are still reported frequently [9,10,11,12]. It may be that especially in older, geriatric patients with comorbidities like osteoporosis the risk of complications such as humeral head necrosis, secondary dislocation or screw loosening due to inferior bone quality is increased.

Similar issues are frequently debated in the case of femoral neck fractures. Whereas osteosynthesis using cannulated screws or similar osteosynthesis systems may be a viable option particularly for nondisplaced fractures, less revision surgeries and good clinical outcomes have led arthroplasty to become the gold standard for geriatric femoral neck fractures [13,14]. Therefore, arthroplasty for PHFs may be favorable especially in older patients as well [8,15]. While results for Hemiarthroplasty (HA) following PHFs in general are inconsistent [16,17,18], studies revealed better clinical results for geriatric patients treated with reverse total shoulder arthroplasty (rTSA) [19,20,21,22]. However, in the aforementioned studies a variety of fracture types were included. For this reason, it can be assumed that when looking at complex intraarticular fractures (type C fractures according to AO) superior findings of arthroplasty may be even more pronounced when directly compared to ORIF [23,24]. Therefore, the purpose of this study was to compare adverse events and clinical outcomes of geriatric PHFs involving the anatomical neck (type C according to AO classification) treated with ORIF using locking plate vs. arthroplasty. We hypothesized that ORIF would result in a higher occurrence of adverse events and inferior clinical outcomes when compared to arthroplasty.

## 2. Methods

This retrospective cohort study was performed in accordance with the Declaration of Helsinki under the approval of the local ethics committee (number 837.503.14/9742). Written informed consent to participate in this study was obtained from all patients.

**Figure 1:**
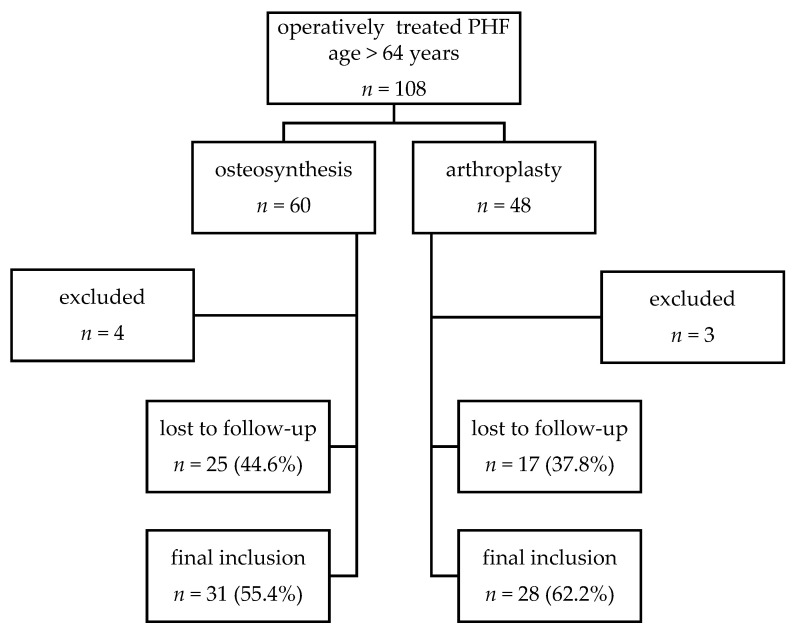
Flow chart of study population recruitment. Proximal humeral fractures (PHF).

Between January 2008 and October 2014 patients with operatively treated PHFs were included if they met the following criteria: PHFs involving the anatomical neck (fracture type C according to the AO classification [23]), age over 64 years, informed consent and minimum clinical follow-up of 1 year. Exclusion criteria were open fractures, pathological fractures, previous operative treatment of the proximal part of the humerus, concomitant ipsilateral fractures of the distal part of the humerus or the elbow joint and severely injured patients with an Injury Severity Score of >16. A total of 108 PHFs met the inclusion criteria. Of those, 7 were excluded due to previous surgeries or concomitant distal fractures of the ipsilateral arm (ORIF *n* = 4; arthroplasty *n* = 3). Twenty-one patients had medical conditions precluding them from clinical follow-up in clinic (ORIF *n* = 12; arthroplasty *n* = 9). Twelve patients had died (ORIF *n* = 8; arthroplasty *n* = 4) and 9 refused to participate (ORIF *n* = 5; arthroplasty *n* = 4). This left 59 patients for inclusion (Figure 1).

Operative Technique

All patients underwent a standardized operative procedure and postoperative rehabilitation protocol. Prior to surgery all patients underwent a computed tomography of the involved shoulder.

Depending on the severity of the fracture and patient factors (e.g., comorbidities, preference of the patient) osteosynthesis using a proximal humeral locking-plate (PHILOS, DePuy Synthes, Umkirch, Germany) or arthroplasty (hemiarthroplasty: GLOBAL FX Shoulder Fracture System, Synthes, Umkirch, Germany; reversed total shoulder arthroplasty: DELTA XTEND System, Synthes, Umkirch, Germany) was chosen for treatment.

In general, ORIF was performed if the fracture was deemed reconstructable and arthroplasty if it was not (comminuted or severely dislocated fractures). All surgical procedures were performed in the beach-chair position, using a deltopectoral approach.

### 2.1. ORIF

Following a standard deltopectoral approach the locking-plate was aligned parallel to the shaft axis (5 mm distal to the tip of the greater tuberosity and 2 mm lateral to the bicipital groove) and fixed to the bone with a cortical screw. After fracture reduction and, if necessary, brief transfixation with K-wires, the plate was fixed by placing the locking-screws into the humeral head. Based on individual assessment of the surgeon, additional fixation of the tuberosities using nonabsorbable high-strength sutures (#2 Fibrewire, Arthrex, Naples, FL, USA) was performed. The wound was then copiously irrigated and closure was obtained in a layered fashion.

Postoperatively, the shoulder was immobilized in a sling. If the tuberosities were repaired, a shoulder abduction pillow (Medi SAK^®^; Medi, Bayreuth, Germany) at 30° was used after two days. Passive range- of-motion exercises started within 2 days after surgery up to 90° of abduction/flexion and limited to 20° of external rotation (if the tuberosities were repaired). This was followed by active-assisted range-of-motion exercises at 3 weeks and unrestricted, active range-of-motion and strengthening exercises at 7 weeks postoperatively.

### 2.2. Arthroplasty

Again, a deltopectoral approach was used. If necessary, the subscapularis tendon was sharply detached from the lesser tuberosity and repaired in all cases following arthroplasty. Based on rotator cuff integrity, treatment with hemiarthroplasty or rTSA was performed. In cases of (pre-) existing rotator cuff defects or non-reconstructable tubercula rTSA was chosen. For rTSA, the glenosphere (38 mm/42 mm) was implanted using a minimum of 2 and up to 4 polyaxial locking-screws. In all cases a cemented monoblock humeral stem was chosen. For proper stability polyethylene inlays ranging from 3 to 9 mm were used after testing for adequate tension with trial implants. If possible, both tuberosities were repaired using suture-cerclages (#2 Fibrewire, Arthrex, Naples, FL, USA).

For hemiarthroplasty, a cemented 140 mm stem was implanted. With regard to achieve proper stability, head diameters between 44 mm and 52 mm were chosen. In cases of hemiarthroplasty, refixation of the tuberosities was mandatory. The wound was then copiously irrigated and closure was obtained in a layered fashion.

Postoperatively, the shoulder was immobilized in a sling for 1 to 2 days and then changed to a shoulder abduction pillow (Medi SAK^®^; Medi, Bayreuth, Germany) at 30° for 4 weeks.

Pain-free, passive range-of-motion up to 60° of abduction/flexion and 20° of external rotation was started on the first postoperative day and was increased after 4 weeks to 90° abduction/flexion and 30° of external rotation. Unrestricted, active range-of-motion and strengthening exercises began at 7 weeks postoperatively.

### 2.3. Clinical Evaluation

For all included patients, electronic patient records were screened for comorbidities (Charlson Comorbidity Index) and preoperative medication, time interval from injury to surgery. Additionally, complications and revisions were documented. All events potentially subject to revision surgery were evaluated as complication. In case of multiple, subsequent revisions in one patient, the main revision was considered. Kaplan-Meier survival analysis was performed to examine and depict survival of the procedure. Failure was defined as revision surgery. Clinical outcomes were assessed by two examiners who were blinded to the type of surgery using the age and gender adjusted Constant Murley Score (CMS%) [25,26] and the Disabilities of the Arm, Shoulder and Hand (DASH) questionnaire [27]. Range-of-motion (flexion, abduction, external rotation) was evaluated using a goniometer. For the CMS, the maximal abduction strength at 90° was obtained using ISOBEX 2.1 dynamometer (CURSOR AG, Bern, Switzerland).

### 2.4. Statistical Analysis

Statistical analysis was performed using SPSS. Descriptive results are given as mean and standard deviation for continuous variables and frequencies and percentages for categorical data. Nominal/categorical data were compared using the Chi-square test or Wilcoxon signed-rank test. For continuous data, the Mann–Whitney U-test or unpaired t-test were used. For subgroup analysis a one way ANOVA was performed. The level of significance was defined as *p* = 0.05. Evaluation of the data was retrospective; an a-priori sample size estimation was therefore not conducted.

## 3. Results

### 3.1. Demographic Data

In this study, a total of 59 patients (mean age: 75.3 ± 5.5 years) with type C PHFs were included. The patients had a mean of 3.3 comorbidities and took 4.5 drugs before injury. In 31 patients (52.4%) ORIF was performed, while 28 patients (47.6%) underwent primary arthroplasty (Hemiarthoplasty *n* = 14, reversed shoulder arthroplasty *n* = 14). The arthroplasty group had significantly more C3 fractures (*p* = 0.002). The mean follow up was 2.7 ± 1.7 years. Regarding demographic data no significant differences were found between groups. However, the arthroplasty group tended to have more comorbidities, thus resulting in a higher Charlson Comobidity Index (Table 1).

### 3.2. Hospital Course, Complications and Revisions

Mean stay at the hospital was 12.1 ± 4.8 days. Patients underwent arthoplasty had a significantly longer stay (13.7 vs. 10.6 days; *p* = 0.001) (Table 2). In addition, they tended to need opioids more often at time of discharge (14.3% vs. 6.5%; *p* = 0.409)

Twelve patients (20.3%) had one or more complications. Significantly more complications were found for the ORIF group (10 patients, 32.6%) when compared to in the arthroplasty group (2 patients, 7.1%) (*p* = 0.023). (Table 2 and Table 3).

A total of 18 revision surgeries were performed in 11 patients (18.6%). The ORIF group revealed significantly more revision surgeries when compared to arthroplasty (29.0% vs. 7.1%; *p* = 0.045). (Table 2) Conversion to arthoplasty following humeral head necrosis was the most common revision surgery in the ORIF group (six patients; 19.6%). Of those, three patients had a simultaneous infection and were therefore revised utilizing a two-stage protocol (first surgery: hardware removal, humeral head resection and antibiotic cement spacer implantation; second surgery: arthroplasty). In the arthoplasty group, 1 revision was related to HA and 1 to rTSA both following dislocation (Table 4).

**Figure 2:**
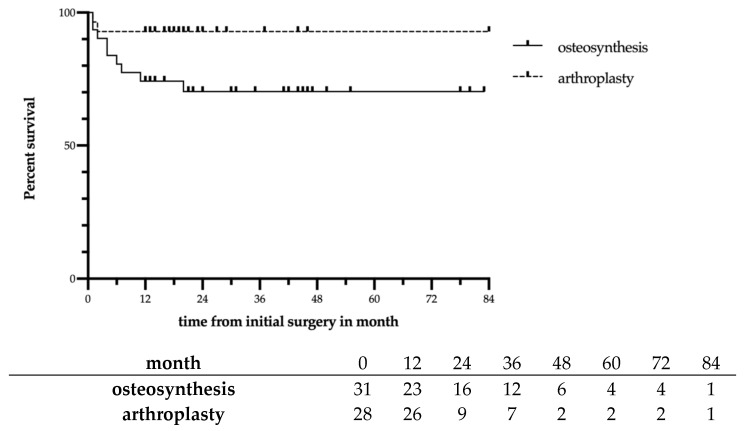
Kaplan-Meier survival curve with number at risk table for the osteosynthesis vs. arthroplasty group with revision surgery defined as failure. The log-rank test indicates a significant difference between the survival curves (*p* = 0.046).

The Kaplan-Meier analysis also indicated a significant difference in time to revision surgery with the ORIF group showing lower survival when compared to the arthroplasty group (*p* = 0.046). All revisions were performed within 20 months of initial surgery (Figure 2).

In addition, eight patients in the ORIF group required elective hardware removal. Therefore, 54.8% of patients in ORIF group and 7.1% in arthroplasty group underwent reoperation.

### 3.3. Clinical Outcomes

Mean age and gender adjusted CMS for the complete cohort of 59 patients was 49.5 ± 27.2 and no significant difference in CMS was detected between groups (Table 5). Patients who underwent revision surgery had a significantly lower CMS (26.3 vs. 54.9; *p* = 0.001). The type of arthoplasty revealed no differences regarding the CMS (HA 47.4 vs. rTSA 51.4; *p* = 0.678)

The DASH Score (mean: 39.6 ± 24.6 pts) demonstrated no significant difference between ORIF and arthoplasty as well as between type arthoplasty (32.8 vs. 45.7; *p* = 0.169) Again, patients who underwent revision surgery had significantly inferior results (58.8 vs. 35.3; *p* = 0.003)

Regarding range-of-motion, the ORIF group had a significantly higher external rotation compared to the arthroplasty group (8.0° vs. 21.3°; *p* = 0.036), whereas no significant difference was found for flexion and abduction (Table 5). Regarding type of arthroplasty no significant difference in range-of-motion was detected.

Fracture severity (according to AO classification) had no significant impact on clinical outcome (Table A1).

## 4. Discussion

The most important finding was that patients who underwent ORIF had a significantly more complications (32.6% vs. 7.1%; *p* = 0.023) and revisions (29.0% vs. 7.1%; *p* = 0.045) compared to patients who were treated with arthroplasty. Therefore, our main hypotheses can be confirmed. In contrast, we found no relevant difference regarding clinical outcome between the two groups.

Knowledge regarding outcomes after osteosynthesis compared to arthroplasty in geriatric PHFs is rare. Recently, Fraser et al. reported in a prospective randomized controlled study the advantage of rTSA over ORIF. They demonstrated a mean Constant Score of 68 for rTSA and 54.6 for ORIF [19]. Both groups reached considerably higher scores compared to the present study. An apparent explanation for the discrepancies between both studies is not at hand. Except for the exclusion of head split fractures in their study, demographic data, surgical techniques and rehabilitation did not differ to a large extend. Unfortunately, comprehensive information regarding comorbidities is lacking in the study of Fraser et al. [19].

Number of revisions in ORIF group reveals strong discrepancies between the two studies. Whereas Fraser et al. reported about 13% revisions, we demonstrated 29.1% (54.8% including elective hardware) reoperations in patients who underwent ORIF. We assume that the selection of fracture type might have had an influence. In addition to 44.4% of C2 fractures, Fraser et al. also included B2 fractures which may ultimately better reflect the reality of trauma care. However, these fracture types differ considerably both biologically and biomechanically. In particular, type C fractures may be inappropriate for osteosyntheses due to (1) a high risk of vascular compromises and (2) less bone stock for screw fixation und thus may result in significantly higher risk of revision surgery [24,28,29]. In fact, humeral head necrosis and screw dislocation was the most common reason for revision in the current study.

A humeral head necrosis in 19.6% in our ORIF group seems considerably higher than described previously [30]. Here, the proximity of the fracture to the humeral head may have impacted the results. Fractures of the anatomic neck have been previously identified as risk factor for humeral head necrosis [24]. Due to fracture complexity and its need of sufficient preoperative diagnostics (CT-scan) and planning (potential need of arthroplasty), the interval from trauma to surgery was quite long in both the arthroplasty (7.6 d) and the ORIF group (5.5 d). There is evidence, that a trauma-to-treatment interval of more than five days is related to a significant increase in complications in ORIF of PHFs [31,32] which may be an explanation for the relatively high rate of humeral head necrosis in this study as well.

Additionally, screw loosening, which was identified as the second most common reason for revision surgery, can be explained by the lack of sufficient bone stock in the current analysis. These findings strongly suggest that particularly type C fractures of geriatric patients pose an increased risk for complications following ORIF.

In contrast, the occurrence of complications and revisions after arthroplasty were comparable with prior data of arthroplasty following PHFs [4,19]. In the current study, both ORIF and arthroplasty groups had significantly lower clinical outcomes following revision surgery. In addition, it should be acknowledged that every revision may have a considerable impact on the physical and psychological condition of patients in general and geriatric patients in particular. Therefore, we consider our results regarding adverse events to be highly relevant for clinical practice.

We found no relevant differences regarding clinical outcomes between ORIF and arthroplasty. Only external rotation was significantly reduced in patients receiving arthroplasty. This corroborates existing knowledge about compromised external rotation particularly after rTSA [33,34]. Here, a loss of infraspinatus function and insufficient compensation of the posterior deltoid plays a role.

Until recently, hemiarthroplasty was the only salvage procedure for unreconstructable PHFs but demonstrated mediocre results which were not superior to conservative treatment [8,17,18]. The demanding surgical technique to restore anatomy and the absolute necessity for sufficient reconstruction of the tuberosities are challenging [16].

For rTSA more encouraging results were published [20,21,22]. In the present study neither number of adverse events nor clinical outcomes were found to differ between prosthesis types (HA vs. rTSA). In contrast, a comparative study by Sebastia-Forcada et al. found significantly better clinical outcomes for rTSA compared to HA in PHF [35]. Probably, our cohorts were too small for a valid statement.

Additionally, one can assume that in mid- to long-term follow-up, the risk of failure following HA will unproportionally rise due to secondary glenoid wear and rotator cuff insufficiency.

Independent of the debate about type of arthroplasty, the substantial higher complication and revision incidents and also recently reported inferior clinical outcome indicate [19] a critical evaluation of osteosynthesis in geriatric PHF, in particular in type c fractures.

Irrespective from current studies, it is still controversial whether conservative treatment is at least equivalent to operative management, even in complex PHFs. The prospective randomized PROFHER study did not find a difference between conservative and operative treatment of 2-, 3- and 4-part fractures [36]. In contrast, Olerud et al. demonstrated significantly better clinical outcomes for ORIF of geriatric displaced three-part fractures when compared to conservative treatment in their prospective randomized controlled study [4]. Again, in this study, revision surgery (13%) after ORIF was much lower compared to the current investigation. The reason for this may again be explained by the different fracture selection. To our knowledge, there are no studies comparing surgical and conservative treatment of type C-fractures. Therefore, an additional group with conservatively treated patients would be of interest.

Our study is limited by its retrospective design. Main limitation is the lack of objective parameters to decide whether an ORIF or arthroplasty was chosen. On the other hand, this reflects the clinical practice. It can be assumed, which is also confirmed by the distribution of fracture types, that the more severe fractures were treated more frequently by arthroplasty. This emphasizes the significantly lower number of adverse events related to arthroplasty group even more. Of course, the disproportion of fracture types might cause bias, but subgroup analysis revealed no influence on rate of adverse events not clinical outcome (see Appendix A).

Due to the long period of inclusion (six years), various surgeons where involved. It might be assumable that arthroplasty was performed by more experienced, specialized surgeons, whereas ORIF done by less experienced surgeons. On the other hand, rather complex fractures were included in this study; therefore, almost all patients were treated by experienced surgeons.

The follow-up rate was relatively low with one of the reasons being the old age of patients that were included (12 patients had died and 21 were not able to attend for follow-up due to severe comorbidities). On the other hand, this is somewhat in the nature of a study that investigates geriatric fractures. Further differences regarding demographic data between groups were found. Arthroplasty group tended to have higher age which may affect the subjective DASH Score due to lower demand in daily life. However, since the age and gender adjusted CMS revealed similar results, this issue may have no major impact. The arthroplasty group tended to have more comorbidities and a higher Charlson Comorbidity Index, which might increase the risk to adverse events.

Despite same inclusion and follow-up periods being chosen, arthroplasty group had an unexpected shorter follow up time. One hypothesis is that the recent trend towards using arthroplasty increasingly in PHF [37] is also reflected in our clinical practice. Time to event analysis indicated that all revisions were performed within 20 months after initial surgery; therefore, the imbalance regarding follow-up time should not have a major impact on the results of this study. Nevertheless, a mean follow-up period of 2.7 years is rather short, particularly for arthroplasty-related complications such as rotator cuff insufficiency, polyethylene wear or aseptic loosening. It can be assumed that the number of complications will rather rise over time in the arthroplasty group. In contrast, we found short-term complications to be more likely in the ORIF group. For this reason, we have to acknowledge that the superior results of arthroplasty may relativize with longer-term follow-up when compared to ORIF. Therefore, we plan a subsequent five year follow up.

The strength of this work includes the strict focus on intraarticular fractures (type C), which is based on biomechanical considerations. Regardless, we were able to follow up a substantial number of patients treated in a standardized manner, referring to patient records, patient-reported outcome measures and functional testing.

## 5. Conclusions

ORIF of type C PHFs in geriatric patients results in significantly more complications and revision surgery when compared to arthroplasty in short- to midterm follow up. Therefore, osteosynthesis of geriatric intraarticular fractures of the proximal humerus must be critically evaluated.

## Figures and Tables

**Table 1:** Demographic data; osteosynthesis vs. arthroplasty.

Variable	Osteosynthesis (*n* = 31)	Arthroplasty (*n* = 28)	*p* *
Sex			
Male	24 (77.4)	23 (82.1)	0.752
Female	7 (22.6)	5 (17.86)	
Age, y	74.1 ± 3.7	76.7 ± 6.9	0.079
Fracture distribution acc. AO	C 1 = 14 (45.2) C 2 = 6 (19.4) C 3 = 11 (35.5)	C 1 = 2 (7.1) C 2 = 4 (14.3) C 3 = 22 (78.6)	0.002 **
Concomitant injury	10 (32.3)	5 (17.2)	0.238
Comorbidities	3.1 ± 2.0	3.4 ± 1.7	0.533
No. medications preoperative	4.6 ± 3.0	4.4 ± 3.3	0.671
Charlson Comorbidity Index (pts.)	3.8 ± 1.5	4.3 ± 1.7	0.460
Follow-up time, y	3.4 ± 1.7	2.0 ± 1.3	0.001 **

Continuous data presented as mean and standard deviation; categorical data as frequencies and percentage (Chi^2^-test) * *p* value for differences between osteosynthesis and arthroplasty; ** significant difference.

**Table 2:** Hospital course, complications and revisions; osteosynthesis vs. arthroplasty.

Variable	Osteosynthesis (*n* = 31)	Arthroplasty (*n* = 28)	*p* *
Time from injury till operation	5.5 ± 4.7	7.6 ± 7.1	0.460
Time in hospital	10.6 ± 5.2	13.7 ± 3.7	0.001 **
Complications	10 (32.6)	2 (7.1)	0.023 **
Revisions	9 (29.0)	2 (7.1)	0.045 **

Continuous data presented as mean and standard deviation; categorical data as frequencies and percentage (Chi^2^-test) * *p* value for differences between osteosynthesis and arthroplasty; ** significant difference.

**Table 3:** Complication during follow up.

Osteosynthesis (*n* = 31)	Arthroplasty (*n* = 28)
*without complication n = 21 (67.4)*	*without complication n = 26 (92.8)*
humeral head necrosis *n* = 6 (19.6)	HA dislocation *n* = 1 (3.6)
infection *n* = 3 (9.8)	rTSA dislocation *n* = 1 (3.6)
intraarticular screw *n* = 2 (6.4)	
posttraumtic omarthritis *n* = 1 (3.2)	
pseudarthrosis *n* = 1 (3.2)	
periosteosynthetic fracture *n* = 1 (3.2)	
rotator cuff deficiency *n* = 1 (3.2)	

Type of major complications in total given as frequencies and percentage; HA hemiarthroplasty, rTSA reversed total shoulder arthroplasty. Of note, a single patient could have had more than one complication. Therefore, total complications do not match up to 100% in the osteosynthesis group.

**Table 4:** Revisions during follow up.

Osteosynthesis (*n* = 31)	Arthroplasty (*n* = 28)
*without revision n = 22 (71)*	*without revision n = 26 (92.8)*
conversion to rTSA *n* = 5 (16.1)	conversion HA to rTSA *n* = 1 (3.6)
singular screw removal *n* = 2 (6.4)	open reduction and “upsizing” *n* = 1 (3.6)
conversion to TSA *n* = 1 (3.2)	
reosteosynthesis *n* = 1 (3.2)	

Type of revision given as frequencies and percentage; HA hemiarthroplasty, TSA total shoulder arthroplasty, rTSA reversed total shoulder arthroplasty.

**Table 5:** Clinical outcome; osteosynthesis vs. arthroplasty.

Variable	Osteosynthesis (*n* = 31)	Arthroplasty (*n* = 28)	*p* *
**DASH (pts)**	39.9 ± 25.7	39.25 ± 24.5	0.922
**Constant Murley Score (pts)**	49.7 ± 29.2	49.4 ± 25.2	0.731
**Flexion °**	88.6 ± 38.9	102.0 ± 24.4	0.394
**Abduction in °**	88.6 ± 29.7	93.0 ± 30.6	0.770
**External rotation in °**	21.3 ± 14.6	8.0 ± 9.2	0.036 **
**Elevation Strength in kg**	1.4 ± 1.5	1.2 ± 1.5	0.533

Continuous data presented as mean and standard deviation; * *p* value for differences between osteosynthesis and arthroplasty; ** significant difference.

## Data Availability

The data presented in this study are available on request from the corresponding author. The data are not publicly available due to ethical principles.

## Appendix A

**Table A1:** Influence of fracture type on outcome.

Type of Fracture	*n*	Complications	*p* *	Revisions	*p* *	DASH (pts)	*p* *	CMS (pts)	*p* *
11 C 1	16 (27.1)	4 (25.0)	0.257	6 (35.3)	0.101	41.4 ± 27.8	0.876	49.6 ± 33.6	0.770
11 C 2	10 (17.0)	2 (20.0)		1 (10.0)		36.2 ± 22.8		55.0 ± 19.9	
11 C 3	33 (55.9)	6 (18.8)		4 (12.1)		39.7 ± 24.1		47.8 ± 26.2	

Continuous data presented as mean and standard deviation; categorical data as frequencies and percentage; * *p* value for differences between variables using Chi-square test/one-way ANOVA.

**Table A2:** Influence of fracture type on outcome in ORIF group.

Type of Fracture	*n*	Complications	*p* *	Revisions	*p* *	DASH (pts)	*p* *	CMS (pts)	*p* *
11 C 1	14	4 (28.6)	0.175	6 (42.9)	0.305	41.8 ± 29.0	0.768	46.9 ± 32.9	0.576
11 C 2	6	2 (33.3)		1 (16.7)		33.0 ± 18.4		61.2 ± 20.8	
11 C 3	11	4 (36.4)		2 (18.2)		41.2 ± 24.2		46.8 ± 28.9	

Continuous data presented as mean and standard deviation; categorical data as frequencies and percentage; * *p* value for differences between variables using Chi-square test/one-way ANOVA.

**Table A3:** Influence of fracture type on outcome in arthroplasty group.

Type of Fracture	*n*	Complications	*p* *	Revisions	*p* *	DASH (pts)	*p* *	CMS (pts)	*p* *
11 C 1	2	0	0.617	0	0.683	38.4 ± 25.0	0.988	68.5 ± 44.6	0.548
11 C 2	4	0		0		41.0 ± 30.8		45.8 ± 16.8	
11 C 3	21	2 (9.6)		2 (9.6)		39.0 ± 24.7		48.3 ± 25.4	

Continuous data presented as mean and standard deviation; categorical data as frequencies and percentage; * *p* value for differences between variables using Chi-square test/one-way ANOVA.
